# Strain Typing of Classical Scrapie by Transgenic Mouse Bioassay Using Protein Misfolding Cyclic Amplification to Replace Primary Passage

**DOI:** 10.1371/journal.pone.0057851

**Published:** 2013-03-05

**Authors:** Katy E. Beck, Leigh Thorne, Richard Lockey, Christopher M. Vickery, Linda A. Terry, Raymond Bujdoso, John Spiropoulos

**Affiliations:** 1 Transmissible Spongiform Encephalopathy Department, Animal Health and Veterinary Laboratories Agency, Addlestone, Surrey, United Kingdom; 2 Department of Pathology, Animal Health and Veterinary Laboratories Agency, Addlestone, Surrey, United Kingdom; 3 Department of Veterinary Medicine, University of Cambridge, Cambridge, United Kingdom; University of Maryland School of Medicine, United States of America

## Abstract

According to traditional murine bioassay methodology, prions must be serially passaged within a new host before a stable phenotype, and therefore a strain, can be assigned. Prions often transmit with difficulty from one species to another; a property termed the transmission barrier. Transgenic mouse lines that over express prion protein (PrP) genes of different species can circumvent the transmission barrier but serial passages may still be required, particularly if unknown strains are encountered. Here we sought to investigate whether protein misfolding cyclic amplification (PMCA), an *in-vitro* method of PrP^Sc^ replication, could be used to replace serial passage of VRQ/VRQ classical scrapie isolates undergoing strain typing in ovine transgenic tg338 mice. Two classical scrapie field isolates that do not readily transmit to wild-type mice underwent bioassay in tg338 mice pre- and post- PMCA and the phenotype of disease in inoculated mice was compared. For one of the sources investigated, the PMCA product gave rise to the same disease phenotypes in tg338 mice as traditional bioassay, as indicated by lesion profile, IHC analysis and Western blot, whilst the second source produced phenotypic characteristics which were not identical with those that arose through traditional bioassay. These data show that differences in the efficiency of PMCA as a strain-typing tool may vary between ovine classical scrapie isolates and therefore suggest that the ability of PMCA to replace serial passage of classical scrapie in tg338 mice may depend on the strain present in the initial source.

## Introduction

Pathogenic isoforms (PrP^Sc^) of host encoded prion protein (PrP^C^) are considered to be the causative agents of transmissible spongiform encephalopathies (TSEs), which include classical scrapie in small ruminants [1], [2]. These misfolded proteins termed ‘prions’ manifest as distinct strains which produce different stable phenotypes following inoculation into wild-type or transgenic mice [3]–[5]. Transmission of prion infectivity between species can be labored, a property termed the transmission barrier, which is often associated with prolonged incubation periods of disease and low attack rates [6], [7]. This phenomenon can also occur between animals of the same species expressing different prion protein genotypes [8]. Serial passage of an isolate in a new host allows prions to stabilize (i.e. to reproduce a characteristic stable disease entity) so that a phenotype, and therefore a strain, can be assigned. One approach to overcome the species barrier has been to develop transgenic mouse lines that over express prion protein transgenes of different genotypes representing various species on a murine PrP ablated background. When the PrP sequence between the TSE source and transgenic mice is matched, transgenic mice can be more sensitive than wild-type mice and succumb to disease with shortened incubation periods [9], [10]. However serial passages may still be required for strain characterization, particularly if previously unrecognized strains are encountered [9], [11].

Using protein misfolding cyclic amplification (PMCA), an *in-vitro* technique able to convert host PrP^C^ to PrP^Sc^ [12], resultant PrP^Sc^ has been shown to be infectious [13] and several studies have verified the biological and molecular strain characteristics of *in-vitro* generated PrP^Sc^ to be analogous with the original PMCA seed [14]–[16]. Although the technique has also been reported to abrogate existing transmission barriers, this sometimes results in the generation of new infectious prions, possibly akin to the process of strain stabilization on serial passage *in-vivo* [14], [17]. If PMCA is indeed able to faithfully replicate strains, as defined by serial passage in a given mouse line, experiment duration could be significantly reduced and there would be a much reduced requirement for the utilization of animals.

In a recent study, cervid PrP^C^ expressed in the brains of Tg(CerPrP) transgenic mice, was used as a substrate to generate chronic wasting disease (CWD) prions using PMCA [14]. The resultant prions were shown to be infectious in Tg(CerPrP) mice and furthermore, they produced unaltered strain characteristics when compared to mice at primary passage infected with the same original tissue. On this basis the aim of the current study was to establish whether PMCA could be used for classical scrapie strain typing studies as a means to replace serial passaging. Specifically, PMCA was used to amplify PrP^Sc^ from scrapie isolates that do not readily transmit to wild-type mice so as to accelerate the strain typing process carried out by murine bioassay. We hypothesized that PMCA of VRQ/VRQ classical scrapie isolates would produce the same disease phenotype on transmission to ovine transgenic tg338 mice overexpressing a VRQ allele on a murine PrP null background as standard bioassay of non-amplified PrP^Sc^ in the same mouse line. For one isolate, mice inoculated with PMCA product reproduced the disease phenotypes observed in mice inoculated with non-amplified product. Conversely, mice inoculated with a second PMCA product exhibited differences in phenotype compared with mice inoculated with corresponding non-amplified material. These data show that differences in the efficiency of PMCA as a strain-typing tool may vary between ovine classical scrapie isolates and therefore suggest that the ability of PMCA to replace serial passage of classical scrapie in tg338 mice may depend on the strain present in the initial source.

## Materials and Methods

### Ethics Statement

All animal work was approved by the Animal Health and Veterinary Laboratories Agency local ethics committee and was carried out in accordance with the Animals (Scientific Procedures) Act 1986 under Home Office project license 70/6310.

### Sample Selection and Preparation

Two positive classical scrapie field sources, PG752/96 and PG676/98, were investigated. Both sources originated from UK sheep of VRQ/VRQ genotype. For each source, aliquots of 10% (w/v) ovine brain homogenates were prepared in sterile saline and denoted inoculum-3 or inoculum-18, respectively. PMCA substrates (PrP^C^) were prepared from unchallenged sheep (VRQ/VRQ PrP genotype) and ovine transgenic tg338 mice (VRQ/VRQ PrP genotype) using fresh, unfrozen brain tissue according to Thorne and Terry [18]. Brain tissue from PrP null (PrP^0/0^) mice was employed in control experiments for this study.

### Mouse Bioassay

Inoculum-3 [10] and inoculum-18 samples had previously been inoculated into tg338 mice (primary [1°] passage). The original inocula that had been used for the bioassay were also used to seed PMCA. Mice were subsequently inoculated with PMCA products (diluted to 10% (w/v) in sterile saline, 20 µl via intra-cerebral route): those inoculated with PMCA amplified inoculum-3 were referred to as PMCA-3 mice whilst mice inoculated with PMCA amplified inoculum-18 were referred to as PMCA-18 mice. Ten tg338 mice aged 6–10 weeks were challenged with each inoculum or PMCA product. Mice were monitored from 30 days post inoculation for pre-determined clinical signs of TSE infection and euthanized using carbon dioxide when clinical endpoint had been reached or intercurrent disease occurred, resulting in significant deterioration, lack of mobility or inability to eat or drink at any time during the monitoring process, as described previously [19]. Brains were removed under sterile conditions and cut parasagittally to give two portions, the smallest of which (approximately one third) was frozen at −80°C for biochemical analysis or sub passage, while the remaining two thirds were fixed in 10% neutral buffered formalin for at least three days at room temperature prior to histological processing. Brains were cut at four coronal levels to reveal caudal medulla, rostral medulla, midbrain, thalamic and frontal levels required for lesion quantification and profiling as detailed below. Tissues were processed and embedded in paraffin wax using routine histological methods. Sections (3 µm) were subsequently mounted on glass slides for Haematoxylin and Eosin (H&E) staining and immunohistochemistry (IHC) as previously described [20].

Following 1° passage of non-amplified inoculum-3 and inoculum-18, brain homogenate (1% w/v) of histopathologically, and where available, clinically positive animals which succumbed to disease with the shortest (S) and longest (L) incubation period each underwent second (2°) passage into a further panel of 10 tg338 mice.

### Lesion Profiling and Immunohistochemistry

Post mortem TSE diagnosis was confirmed based on the presence of characteristic neuropil vacuolation within the brain on H&E stained sections. Lesion severity in TSE positive samples was further semi-quantified using an established method [19], where the degree of TSE specific vacuolation in pre-determined neuroanatomical areas can be scored and plotted graphically to produce ‘lesion profiles’ which are specific to a given host adapted TSE strain [21]. The mean profile for each inoculum was calculated from at least five or more clinically and histopathologically positive mice. For IHC, samples were labelled with rabbit polyclonal antibody Rb486, using a standard method [22].

### Serial Protein Misfolding Cyclic Amplification (PMCA)

The serial PMCA procedure was performed as described previously [18]. In brief, brain tissues were homogenized at 10% (w/v) in either saline or in PMCA conversion buffer. Homogenates were diluted 1/10, in PMCA substrate (10^−2^ final dilution) then 100 µl placed in a 200 µl PCR tube and subjected to repeat cycles of alternating periods of sonication (40 s; 250W power) and incubation (29 min 20 s), performed at 37°C using an ultra-sonic water bath (misonix S4000) and a deep well microtitre plate horn. After the first 24 h round (48 cycles) PrP^C^ was replenished by diluting the samples 1/1000 in fresh brain substrate and a second 24 h round of PMCA was initiated. The process was continued for another two 24 h rounds diluting the samples 1/1000 between each round. By round four the original tissue had been diluted out to 10^−11^. PMCA products were then stored at −20°C until used for bioassay or analyzed.

### Sample Analysis by Enzyme Immunoassay (EIA)

Pre- and post- PMCA, samples were analyzed by EIA (IDEXX Herdchek BSE/scrapie EIA antigen test kit) using a modified procedure. Samples were diluted 1∶5 in kit homogenization buffer (designated as neat in the dilution curves with further dilutions achieved using the same buffer) then mixed 4∶5 with kit plate diluent. Each sample (100 µl) was applied to the test plate and incubated for 180 min at 26°C. After removal of excess reagents using wash buffer 1, bound sample was incubated with conditioning buffer for 10 min at 26°C, washed again (wash buffer 2) to remove excess reagent and incubated with an anti-PrP horseradish peroxidase conjugated antibody for 90 min at 26°C. After removal of excess reagents using wash buffer 2, visualization of bound PrP^Sc^ was achieved using 3,3′,5,5′-tetramethylbenzidine (TMB) substrate and measured at 450 nm using a reference filter at 620 nm (Perkin Elmer Envision 2104 multi-label reader). Amplification was determined by comparison with pre-amplification reference samples.

### Analysis of PMCA Products by Western Blot

Samples were digested using proteinase K (PK) (100 µg/ml) in the presence of 0.04% (w/v) sodium dodecylsulphate (SDS) for 60 min at 50°C with continuous agitation. Digested samples were mixed with an equal volume of sample buffer [Laemmli sample buffer (Bio-Rad) supplemented with 2% (w/v) SDS and 5% (v/v) 2-mercaptoethanol] and heated at 100°C for 5 min before storage at −20°C. Proteins in 15 µl of sample were separated on 12% Bis-Tris gels (Critterion; Bio-Rad) before electro-transfer to activated PVDF membrane. Unbound membrane was blocked using 5% (w/v) BSA in phosphate buffered saline (PBS) supplemented with 0.1% (v/v) tween20 (PBST). Bound PrP^Sc^ was labeled with anti-PrP Sha31 monoclonal antibody [23] conjugated to biotin and streptavidin peroxidase and visualized by chemiluminescence (ECL; Amersham).

### Analysis of Ovine and Murine Brain Tissues by Western Blot

Samples were homogenized, 20% (w/v), in homogenization buffer (supplied by manufacturer, BioRad TeSeE assay), digested for 10 min at 37°C with an equal volume of PK solution (20 µl/ml) then clarified using buffer B. Precipitated proteins were centrifuged and the resultant pellet was solubilized in sample buffer prior to heating at 100°C for 5 min. Following a second centrifugation to remove non-soluble material, supernatant was stored at –20°C until required. SDS-PAGE and immunoblotting was performed as described above.

## Results

### Generation and Analysis of *in-vitro* Amplified Ovine Scrapie PrP^Sc^

**Figure 1:**
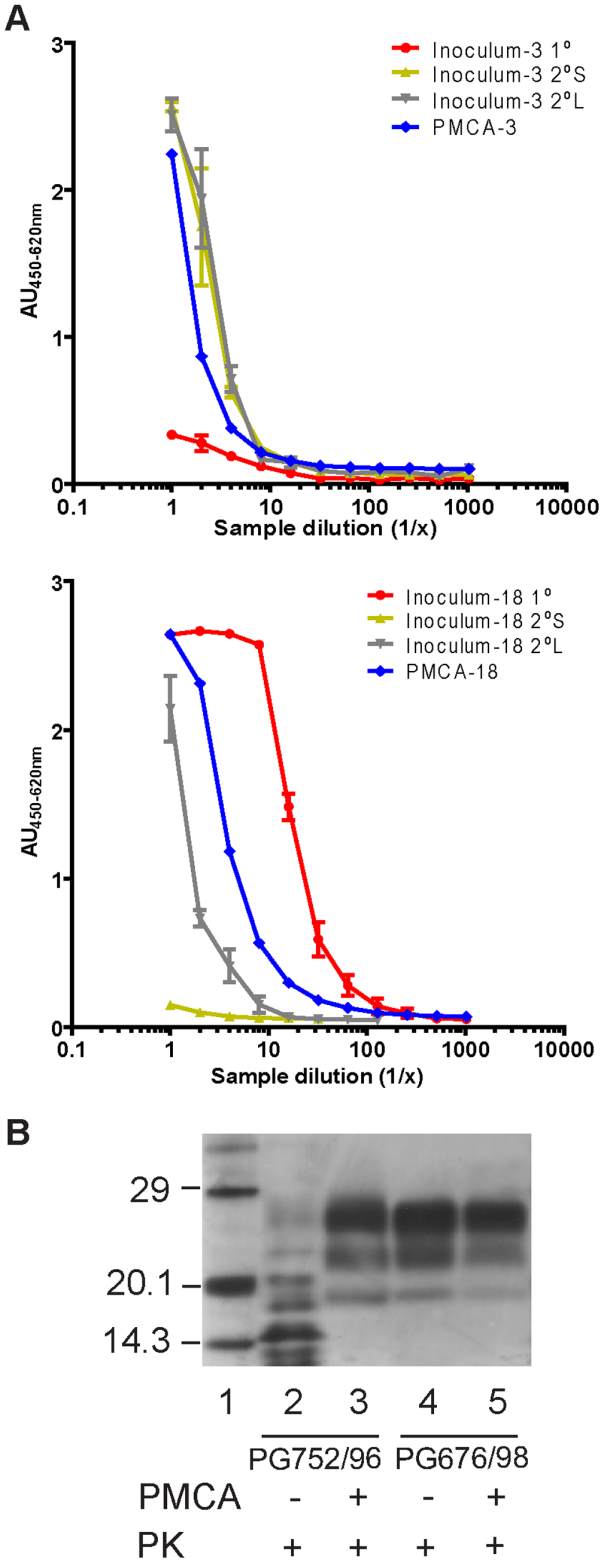
Amplification of ovine PrP^Sc^ using different VRQ/VRQ substrates. (a) EIA titration of PMCA inocula as determined using the IDEXX Herdchek assay compared with corresponding non-amplified inocula. Results represent the mean of three experiments ±SEM. (b) Comparison of original ovine tissues (Lanes 2: PG752/96 and 4: PG676/98) extracted according to Bio-Rad TeSeE Western blot protocol and corresponding PMCA product of the same tissues (Lanes 3: PMCA-3 and 5: PMCA-18) extracted according to the PMCA Western blot protocol. Samples were further diluted in Laemmli sample buffer to achieve visible molecular weight profiles.

*In-vitro* amplification of ovine scrapie by PMCA was demonstrated previously using TSE-free ovine brain homogenates as a source of PrP^C^ [18]. In this current study brain homogenates prepared from mice transgenic for the ovine VRQ *PRNP* gene (tg338 mice) were used as substrates for PMCA. We amplified classical scrapie isolates in these substrates to determine whether serial passage in mice normally required for strain identification can be replaced. A comparison of the efficiency of ovine and tg338 substrates to support amplification of PrP^Sc^ *in-vitro* indicated that amplification efficiency of PrP^Sc^ in tg338 substrates over four PMCA rounds was similar to that amplified in ovine brain tissue (data not shown). In order to be able to compare incubation period data for inocula generated from PMCA products with corresponding inocula from primary and secondary brain homogenates, relative titres were analysed by EIA (IDEXX Herdchek BSE/scrapie EIA antigen test kit) (Figure 1a). For the PMCA inoculum derived from inoculum-3 (PMCA-3), relative PrP^Sc^ quantities were very similar compared with corresponding secondary short (2°S) and long (2°L) sub-passages of the same isolate used for traditional bioassay as shown by similar dilution curves. Notably, a considerably lower PrP^Sc^ quantity was observed with the corresponding original inoculum (inoculum-3) as shown by the lower dilution curve. The PMCA inoculum derived from inoculum-18 (PMCA-18), showed PrP^Sc^ quantities approximately 4 times greater than 2°L inoculum and considerably greater than 2°S inoculum originating from the same tissue. PMCA-18 inoculum had approximately 5 times less PrP^Sc^ than the original inoculum-18.

Western blot analysis (Figure 1b) was used to compare the PrP^Sc^ profiles pre- and post- amplification of the two scrapie isolates. PK resistant PrP polypeptides (PrP^res^) derived from the original ovine tissue of sample PG752/96 (lane 2) did not display the usual 3 band pattern and could not be resolved despite repeated attempts, an observation also made by other laboratories [10]. The lower molecular weight bands observed may represent over digestion of PrP^Sc^ due to diminished PK resistance of this scrapie isolate. Previous immunohistochemical analysis of the ovine brain had confirmed a classical scrapie PrP^Sc^ deposition profile. Interestingly the corresponding PMCA product for PG752/96 gave rise to a three-band pattern (lane 3) indistinguishable from a non-amplified classical scrapie (lane 4). It thus appears that the phenotype of the *in-vitro* amplified PrP^Sc^ from isolate PG752/96 is distinct from the original sample. It is possible that *in-vitro* manipulation might have induced a phenotypic alteration of the PrP^Sc^, subsequently identified on transmission to mice or indeed that PMCA amplified a strain that was not the major strain present in the brain. On gross observation there was no alteration of the molecular profile of the second scrapie isolate used in this study, sample PG676/98, after amplification in mouse tg338 substrate (lanes 4 and 5).

To confirm that residual infectivity of the initial seed in the final PMCA product was absent, PMCA was performed using a PrP^0/0^ brain homogenate substrate. Amplification of PrP^Sc^ is not supported in PrP^0/0^ substrate therefore any infectivity detected by bioassay in the final product would be a result of prions from the original seed. PMCA product using PrP^0/0^ substrate was negative for PrP^Sc^ following biochemical (EIA) analysis and did not induce TSE in tg338 mice maintained up to 700 days post challenge as assessed by histopathology and IHC (data not shown).

### Incubation Periods and Attack Rates Following Bioassay of PMCA Products in tg338 Mice

**Figure 2:**
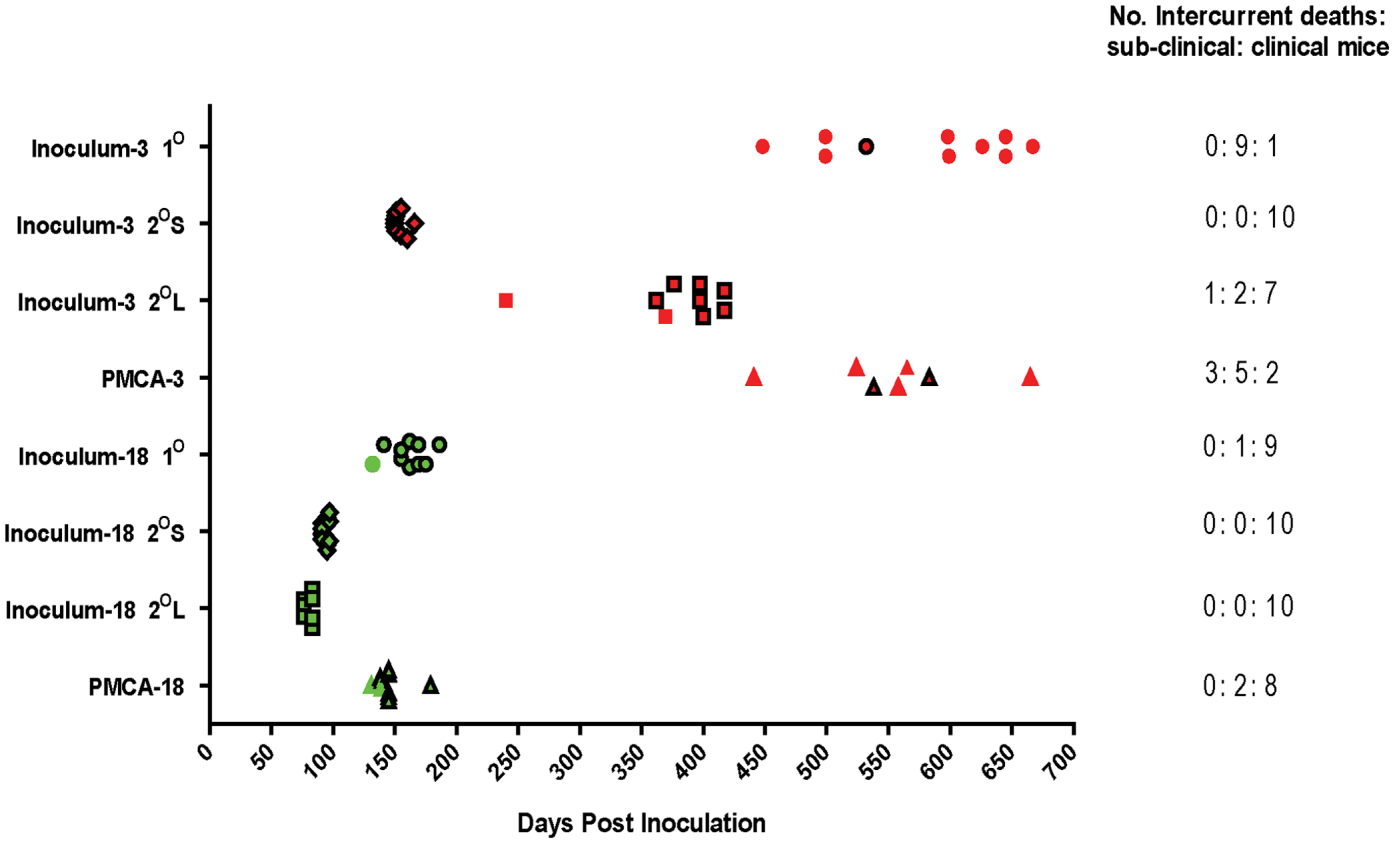
Incubation periods and attack rates of mice inoculated with non-amplified PrP^Sc^ and corresponding PMCA products. Each inoculum was inoculated into 10 tg338 mice, each symbol representing an individual mouse. All TSE positive (histopathological and/or IHC positive) mice are included. Mice that died from intercurrent disease are not shown in the plot. Clinically positive mice are indicated by a black outline and were used to calculate incubation periods. Circles indicate 1°, diamonds indicate 2°S and squares indicate 2°L passage of non-amplified ovine PrP. Triangles indicate mice inoculated with PMCA product. Red symbols denote mice inoculated with products originating from PG752/96 whilst green symbols denote mice inoculated with products originating from PG676/98. The number of mice that died due to intercurrent disease, the number of sub-clinical mice and the number of clinically positive mice are indicated next to each inoculum.

Inoculum-3 (PG752/96) and inoculum-18 (PG676/98) were originally selected as they gave rise to prolonged incubation periods that when inoculated into tg338 mice, were reduced on subsequent passage [10]. The incubation periods and attack rates of TSE positive mice for each inocula i.e. those showing TSE specific vacuolation and/or PrP^Sc^ deposition, are presented in Figure 2. The number of clinically positive mice inoculated with either PMCA product was comparable with primary (1°) passage of the corresponding non-amplified inocula and for both sources this equated to fewer mice than on corresponding secondary (2°) passages. Inoculum-3 previously transmitted poorly to wild-type strains of mice (C57BL/6, RIII and VM) [10] but transmitted to tg338 mice with 100% attack rate with a long incubation period. The mean incubation period of clinically positive mice challenged with the PMCA product derived from inoculum-3 (PMCA-3 challenged mice) was 561±31.8 days post inoculation (dpi), (mean ± StDev) which was similar to inoculum-3 1° mice (532 dpi), but prolonged compared to the second passage of inoculum-3 mice with the shortest (2°S) and longest (2°L) incubation periods (154±5.3 and 395±20.2 dpi, respectively). Inoculum-18 also transmitted to tg338 mice with 100% attack rate but with a much shorter incubation period. Nevertheless the same trend was observed where the mean incubation period for mice challenged with the PMCA product derived from inoculum-18 (PMCA-18 challenged mice) was similar to inoculum-18 1° mice (148±13.1 dpi compared to 164±13.0 dpi). Subsequent incubation periods of second passages of inoculum-18 (2°S and 2°L) were shorter; 94±2.7 and 80±3.6 dpi, respectively. Thus the incubation period was not shortened by *in-vitro* replication of PrP^Sc^ in tg338 substrate to the same extent as serial passage in mice, despite EIA analysis revealing relative PrP^Sc^ titres similar to (PMCA-3 inocula) or greater than (PMCA-18 inocula) corresponding 2°S and 2°L passage inocula, respectively (Figure 1a).

### Lesion Profiling Following Bioassay of PMCA Products in tg338 Mice

**Figure 3:**
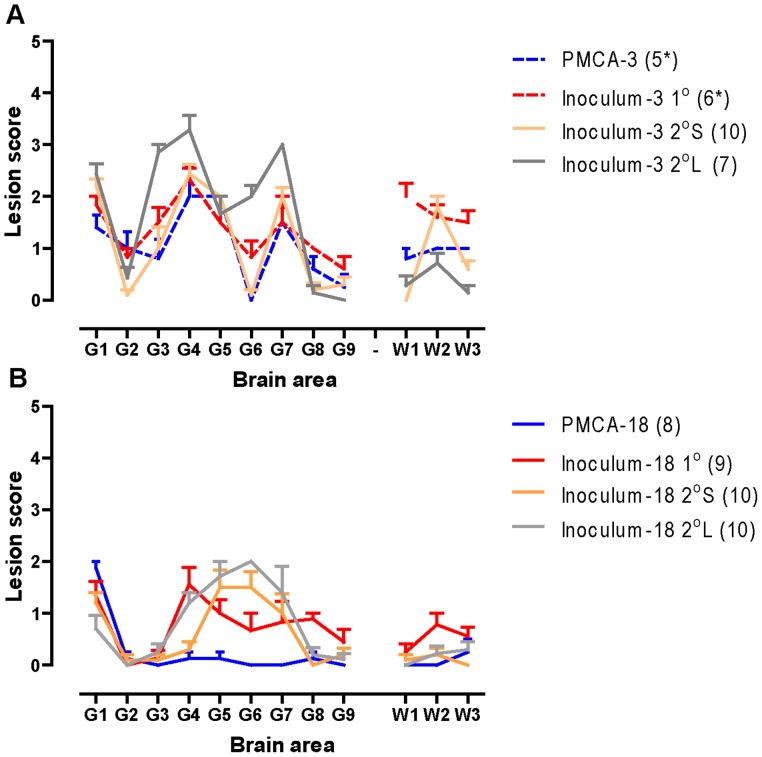
Lesion profiles of tg338 mice inoculated with non-amplified ovine PrP^Sc^ and PMCA product. Lesion profiles were constructed from mice that were clinically and histopathologically positive. The number of mice used to construct each profile is shown in parentheses. (a) Lesion profiles of inoculum-3 and corresponding PMCA-3 mice. (b) Lesion profiles of inoculum-18 and corresponding PMCA-18 mice. (*) indicates that lesion profiles from five clinically and histopathologically positive mice were not available so the profile was constructed from all histopathologically positive mice, irrespective of clinical status for indication only.

The lesion profiles for inoculum-3 challenged mice, sub-passages and PMCA-3 mice are shown in Figure 3°. Profile designations (in brackets) are provided to allow discrimination between profiles within summary Table 1. Lesion profiles of inoculum-3 1°, 2°S and PMCA-3 challenged mice were comparable (Profile 3-a) whilst the lesion profile for 2°L mice differed (Profile 3-b).

**Table 1:** Summary table of results for mice challenged with non-amplified and PMCA amplified inoculum-3 and inoculum-18.

Mouse group	Incubation period (dpi) (Mean ± StDev)	Lesion profile	PrP^Sc^ deposition pattern	Molecular profile§
Inoculum-3 1°	532	Profile 3-a	Apl_338_ii or Apl_338_ii and P_338_ mix	Discrete banding, single non-glycosylated band (18.6 kDa)
Inoculum-3 2°S	154±5.3	Profile 3-a	Apl_338_ii and P_338_ mix	Diffuse banding, single non-glycosylated band (17.1 kDa)
Inoculum-3 2°L	395±20.2	Profile 3-b	Apl_338_ii * or Apl_338_ii and P_338_ mix (single mouse)	Discrete banding, single non-glycosylated band (18.4 kDa)
PMCA-3	561±31.8	Profile 3-a	Apl_338_ii or Apl_338_ii and P_338_ mix	Either: discrete or diffuse banding, single non-glycosylated band (18.3 or 17.1 kDa); or diffuse banding, double non-glycosylated band (17.2 and 18.2 kDa)
Inoculum-18 1°	164±13.0	Profile 18-a	G_338_ii and Apl_338_ii mix	Lower non-glycosylated band (18.9 kDa)
Inoculum-18 2°S	94±2.7	Profile 18-b	G_338_ii * and Apl_338_ii mix	Lower non-glycosylated band (19.2 kDa)
Inoculum-18 2°L	80±3.6	Profile 18-b	G_338_ii * and Apl_338_ii mix	Lower non-glycosylated band (18.9 kDa)
PMCA-18	148±13.1	Profile 18-c	Pattern showing similarities with G_338_ii and Apl_338_ii	Higher non- glycosylated band (19.8 kDa)

Incubation period data (days post inoculation; mean ± StDev), lesion profile, PrP^Sc^ distribution pattern and Western blot profile are summarized for each group of mice. See text for details.

* Predominant PrP^Sc^ deposition pattern indicated.

§ Molecular masses represent the mean value for corresponding Western blot bands shown in Figure 6.

The lesion profiles for inoculum-18 challenged mice, sub-passages and PMCA-18 mice are shown in Figure 3b. The profiles for inoculum-18 2°S and 2°L mice were comparable (Profile 18-b), but differed from the 1° profile (Profile 18-a). The profile of PMCA-18 challenged mice did not resemble either of the profiles derived from inoculum-18 (Profile 18-c).

### Immunohistochemistry for PrP^Sc^ in Murine Brains

IHC for the presence of accumulated PrP^Sc^ was performed on histopathologically positive inoculum-3 and inoculum-18 challenged mice. The type and neuroanatomical distribution of PrP^Sc^ deposition in these mice was compared to that observed in the brains of mice challenged with the corresponding PMCA product.

**Figure 4:**
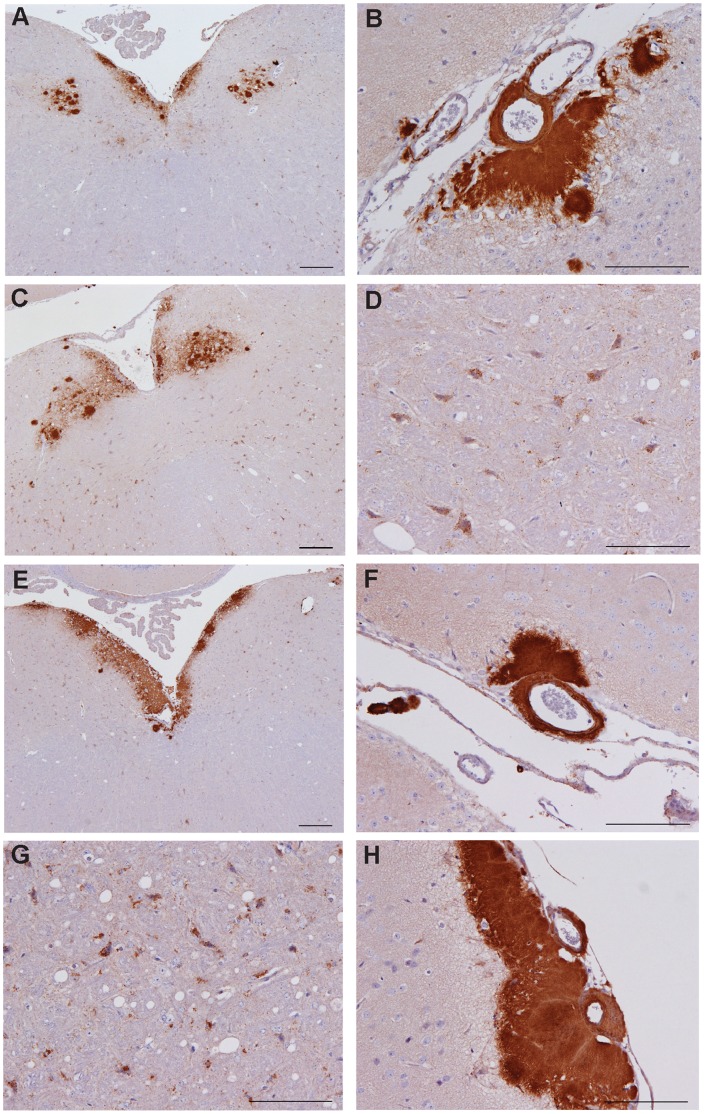
PrP^Sc^ distribution patterns in inoculum-3 and PMCA-3 mice. Patterns identified in inoculum-3 mice are shown in (a–d). The Apl_338_ii pattern is shown in (a); aggregates and plaques in the dorsal medulla and (b); perivascular, lepto-meningial plaques. (c) and (d) represent a mixture of Apl_338_ii and P_338_ coexisting in individual mice: (c) shows the aggregates and plaques of the Apl_338_ii pattern in the dorsal medulla whilst (d) shows intra-neuronal labeling consistent with P_338_ at higher magnification in the same medulla. Patterns identified in PMCA-3 mice are shown in (e) and (f), (Apl_338_ii), and (g) and (h), (Apl_338_ii and P_338_ mixed). Scale bars represent 200 µm (a, c, e) or 100 µm (remaining images).

Mice challenged with inoculum-3 primary (1°) presented two PrP^Sc^ deposition patterns that have been previously reported (Figure 4, a–d), Apl_338_ii, characterised by aggregates and plaques (Figure 4a and b) [10] and P_338,_ characterised by intraneuronal, intraglial and punctate labeling in the neuropil (Figure 4d) [9], [24]. Of the 10 mice analyzed, five exhibited the Apl_338_ii pattern only (Figure 4a and b) and five showed a mixture of Apl_338_ii and P_338_ patterns (Figure 4c and d, respectively). The relative proportion of the two patterns between individual mice was variable. All 2^o^S mice showed a mixture of two patterns, Apl_338_ii and P_338_, with P_338_ features appearing more extensive. 2^o^L mice exhibited the Apl_338_ii pattern, except for a single mouse where an Apl_338_ii and P_338_ mixture was identified with the Apl_338ii_ pattern being dominant. PMCA-3 challenged mice exhibited PrP^Sc^ deposition patterns that were entirely consistent with inoculum-3 1°: three of the six mice analyzed showed Apl_338_ii only (Figure 4e and f) whilst three showed Apl_338_ii and P_338_ mixtures (Figure 4g and h). Where mixtures were observed, the relative proportion of the two patterns between individual mice was variable.

**Figure 5:**
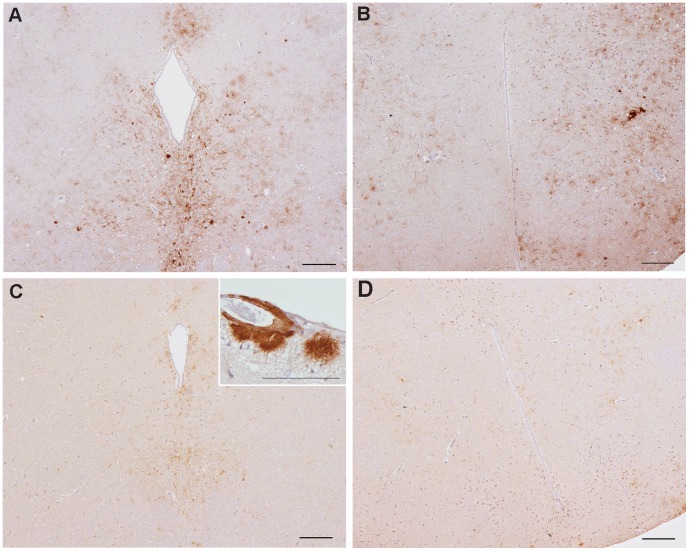
PrP^Sc^ distribution patterns in inoculum-18 and PMCA-18 mice. The distribution of PrP^Sc^ in the midbrain raphe and hypothalamus of inoculum-18 mice are shown in (a) and (b), respectively, and in PMCA-18 mice in (c) and (d), respectively. Perivascular, lepto-meningial plaques observed in PMCA-18 mice are shown in (c inset). Scale bars represent 200 µm or 100 µm (c inset).

All mice challenged with inoculum-18 1° presented a mixture of two possible patterns, exhibited to varying degrees between individual animals. The first pattern was compatible with G_338_ii which is characterised by fine granular deposits of PrP^Sc^, the intensity of which can vary focally to give rise to denser deposits and small aggregates [9]. Elements of Apl_338_ii [10] were also observed. G_338_ii was the predominant pattern observed in all inoculum-18 2°S or 2°L mice. PMCA-18 challenged mice showed a combination of granular and aggregated PrP^Sc^ deposition types similar to those observed in inoculum-18 mice although differences were also noted. In the midbrain of inoculum-18 1° mice, diffuse granular deposition was observed throughout the tissue whilst aggregates were located along the raphe which radiated bilaterally from the midline (Figure 5a). In comparison, in PMCA-18 mice, granular deposition was generally only observed ventral to the aqueduct, a feature of G_338_ii after serial passage, accompanied by a reduction in aggregates within the raphe (Figure. 5c). The intensity of granular deposition was greatly reduced in PMCA-18 mice compared to inoculum-18 1° mice (compare Figure 5b and d), which also reflects the low intensity lesion profile associated with PMCA-18. This was not due to preclinical stage disease as all animals examined were clinically positive. In several PMCA-18 mice, meningial plaques, a characteristic of Apl_338_ii, were also observed (Figure 5c inset). Such formations were not observed in inoculum-18 mice.

### Western Blot for PK Resistant PrP^Sc^ in Murine Brains

**Figure 6:**
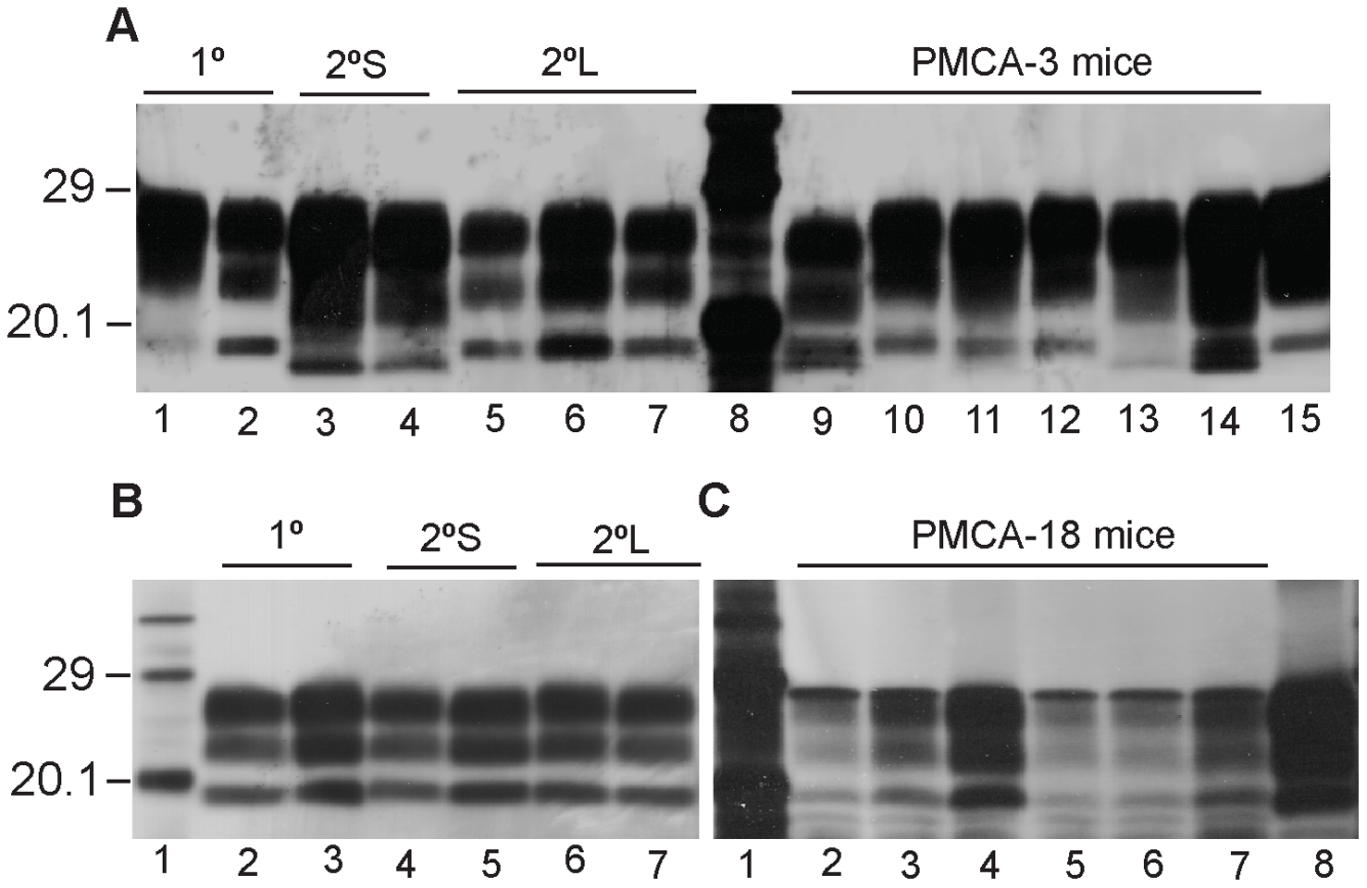
Western blot of PrP^Sc^ from tg338 mice challenged with PMCA products. Inoculum-3 1°, 2°S and 2°L mice are shown in lanes 1–7∶1°, lanes 1 and 2; 2°S, lanes 3 and 4; 2°L, lanes 5 to 7. Lanes 9–14 show mice inoculated with PMCA-3. Lane 15 is loaded with PMCA product prior to inoculation. (b) Inoculum-18 1°, 2°S and 2°L mice are shown: 1°, lanes 2 and 3; 2°S, lanes 4 and 5; 2°L, lanes 6 and 7. (c) Mice inoculated with the PMCA-18 are shown in lanes 2–7. Lane 8 is loaded with PMCA product prior to inoculation. Samples were further diluted in Laemmli sample buffer to achieve visible molecular weight profiles. Remaining lanes show molecular mass markers.

Two distinct molecular profiles of PrP^Sc^ extracted from tg338 mice inoculated with inoculum-3, as shown by Western blot, were observed following secondary passage (Figure 6a) [10]. Primary passaged PrP^Sc^ from inoculum-3 displayed three bands that were indistinguishable from the bands observed from 2°L mice (Figure 6a). However, the non-glycosylated bands from 2°S inoculated mice gave a lower molecular mass of 17.1 kDa compared to 18.6 kDa for the 1° passage mice whilst mono−/di-glycosylated bands were diffuse by comparison. Mice challenged with the PMCA-3 product exhibited PrP^Sc^ of three distinct profiles; mice in lanes 10, 11 and 12 shared similar profiles that were consistent with those observed for the 1° and 2°L passaged mice, although the sample in lane 11 exhibited a slightly lower molecular mass for all three bands. One mouse (lane 13) presented a similar profile to 2°S mice. However, PrP^Sc^ from two mice, shown in lanes 9 and 14, displayed two lower molecular mass bands of 17.2 kDa and 18.2 kDa. Together with the diffuse nature of the mono−/di-glycosylated bands for these samples, this may represent a mixture of the two previously described molecular profiles. The original PMCA product of PG752/96 (lane 15) displayed band patterns consistent with the 1° and 2°L profiles.

Western blot profiles of mice inoculated with inoculum-18 1°, 2°S and 2°L passages (Figure 6b) are shown with the PrP^Sc^ profiles of PMCA-18 challenged mice (Figure 6c). Results show that the profiles of inoculum-18 challenged mice (1°, 2°S and 2°L) share the same electrophoretic mobility (Figure 6b). Mice inoculated with PMCA-18 product shared a molecular profile which was distinct from that observed in inoculum-18 challenged mice and also from the PMCA product prior to inoculation. Inoculum-18 mice and PMCA-18 product prior to inoculation had a non-glycosylated band of 18.9 kDa whereas PMCA-18 challenged mice had a non-glycosylated band of 19.8 kDa. Additional lower molecular mass bands associated with PMCA-18 mice are thought to represent a degree of degradation of the samples, possibly resulting from storage.

### Comparison of IHC and Western Blot Profiles

For inoculum-3 the molecular profiles on Western blot generally correlated with a distinct IHC pattern. In mice where only the Apl_338_ii pattern was observed, the molecular profiles corresponded to the single three band pattern with a higher molecular mass non-glycosylated band. However, mice that exhibited mixtures of Apl_338_ii and P_338_ patterns showed Western blot profiles with either a single lower molecular mass non-glycosylated band or two non-glycosylated bands. The mouse shown in lane 1 was an exception showing a mixed IHC profile but a single higher molecular mass non-glycosylated band. The lower molecular mass non-glycosylated band associated with mixtures of Apl_338_ii and P_338_ patterns has been previously observed in tg338 brains exhibiting P_338_ [24].

Inoculum-18 challenged 1°, 2°S and 2°L mice presented the same molecular profile despite the presentation of a mixture of two possible IHC patterns (G_338_ii and Apl_338_ii) in 1° passage mice and the predominance of a single pattern (G_338_ii) in 2°S and 2°L mice. The different molecular profile exhibited by PMCA-18 mice was consistent with the differences in IHC pattern also observed in these mice.

A summary of each phenotypic parameter for mice challenged with non-amplified and PMCA amplified inoculum-3 and inoculum-18 is given in Table 1.

## Discussion

Here we have successfully amplified ovine prions from scrapie isolates *in- vitro* and showed that they can be subjected to conventional prion strain typing. We demonstrated that brain homogenates from mice transgenic for the ovine *PRNP* gene (tg338) could be used as a substrate for PMCA consistent with previous reports [25] and that the PMCA products from two VRQ/VRQ ovine scrapie isolates infected tg338 mice.

Results for scrapie source PG752/96 indicated that PMCA-3 was able to faithfully amplify the same TSE phenotypes as 1° passage of traditional bioassay, as indicated by lesion profile, IHC analysis and Western blot. These results are in accordance with Green et al. [14] whereby the bioassay of a CWD PMCA product into cervid transgenic mice gave rise to a disease phenotype comparable with the 1° passage via the traditional bioassay.

Whilst the incubation period of inoculum-3 on 2° passages was shortened compared to 1° passage, the incubation periods of PMCA-3 challenged mice remained comparable with 1° passage. A similar trend in incubation period data was also observed for inoculum-18. However, results of EIA titrations indicated that the titres of the PMCA products in the current study were not the cause of disparity. Indeed a previous study reported a prolonged incubation period of products derived from PMCA reactions using beads (PMCAb) [26] seeded with synthetic scrapie strains compared to standard bioassay secondary and tertiary passages with the same strain, in Syrian golden hamsters [27]. It has been proposed that this may represent a reduction in “fitness” of PMCA products to replicate in a cellular environment where fragmentation mechanisms differ to the *in-vitro* approach [28]. We have also previously reported that IHC patterns consistent with known, stabilized classical scrapie strains can be identified in individual wild-type mice on 1° passage of field scrapie sources, before the traditional bioassay strain-typing parameters of lesion profile and incubation period, which rely on mean values of a group of observations, are stabilized [7]. Indeed, in the current study the 2°S passage of inoculum-3 gave mixed PrP^Sc^ distribution patterns of Apl_338_ii and P_338_ where P_338_ was the dominant pattern. In turn the incubation period of disease in these mice was consistent with P_338_ (approximately 150 days), rather than the longer incubation period (approximately 350 days) associated with Apl_338_ii. Whilst it could be expected that the mice would succumb to the agent with the shortest incubation period [29] this nevertheless demonstrates that the Apl_338_ii pattern was also propagating at this time point, though this could not be determined by incubation period and lesion profile analysis only.

Isolate PG676/98 (inoculum-18) revealed two possible phenotypes, one of which gave a granular pattern on IHC analysis of 1° mice akin to G_338_ii, and one in which aggregates were predominant similar to Apl_338_ii. The granular pattern was predominantly exhibited by both 2°S and 2°L mice with a predicted incubation period of approximately 100 days [9]. Although the incubation period of PMCA-18 challenged mice was similar to inoculum-18 1° mice, the lesion profile and Western blot profile presented a phenotype that was not consistent with inoculum-18 or its sub-passages and remains to be elucidated. The Western blot profile of the PMCA product prior to inoculation into mice was consistent with the inoculum-18 1°, 2°S and 2°L Western blot profiles suggesting that a specific adaptation may have occurred on transmission to mice. In turn, IHC revealed subtle differences between mice challenged with non-amplified or PMCA amplified products although common phenotypes were observed. Serial passages of PMCA-18 could be useful in further stabilization and identification of the strain(s) amplified.

It is plausible to consider that differences in phenotype of PMCA-18 challenged mice compared with inoculum-18 1°, 2°S and 2°L mice may have resulted from a lower number of amplification rounds during the PMCA protocol in comparison with other laboratories [14]. In a previous inter-species ‘hamster to mouse’ PMCA study, mice were inoculated with PrP^Sc^ generated after successive rounds of PMCA using a hamster seed and a murine substrate. Results demonstrated that six rounds of serial PMCA were required for stabilization of the new strain [17]. Preceding PMCA round products gave rise to incomplete attack rates and prolonged incubation periods despite the fact that mice were inoculated with equal amounts of PrP^Sc^ as determined by Western blot. This suggests that PMCA was mimicking inter-species strain adaptation as observed *in-vivo*. In the current study there were no inter-species transmissions with respect to the prion protein since both substrate and seed were ovine and of the same genotype. However, the effect of differences in the presence or absence of specific co-factor molecules between brain homogenates from different species on the conversion process is not known, hence there may still have been a process of ongoing adaptation in our system aside from the prion protein that may have stabilized with a greater number of PMCA rounds. An alternative supposition may be that a greater number of PMCA rounds could increase the propensity for “mutations” to arise or spontaneous conversion of PrP^C^ to PrP^Sc^ [18]. Therefore in the PMCA system used in the current study, the number of rounds was chosen to ensure that only specific amplification occurred.

Our data lend further support to evidence that *in-vitro* methods of PrP^Sc^ generation can result in unique disease phenotypes [14], [17], [30], [31], although this is more often observed following cross-species transmissions. Recent studies [27], [28] have suggested that if individual prion strains indeed comprise of a variety of conformers as previously hypothesized by Collinge et al. [32] then changes in the replication environment may evoke selective amplification of particular sub-populations of prions with specific features favorable to that environment. Indeed it has been demonstrated that PMCAb can result in changes in strain specific elongation rates and glycoform ratios consistent with convergent evolution of PrP^Sc^ properties during PMCA [28]. Moreover it has also been shown that changes in glycosylation status of PrP^C^ substrate and presence/absence of RNA co-factors in PMCAb reactions can result in selective amplification of prion sub-populations [27]. Hence an alternative hypothesis for the difference in phenotype of PMCA-18 and inoculum-18 challenged mice is that PMCA may have induced changes in PrP^Sc^ properties owing to the differing replication environment compared to *in-vivo* bioassay, and/or due to these changes in environment, PMCA may have selectively amplified a different sub-population of PrP^Sc^ to those that propagated during bioassay.

The ability of PMCA to reproduce the phenotypes observed following mouse bioassay may depend on the type of prion being amplified. It is possible that TSEs that reportedly comprise of a single agent, such as bovine spongiform encephalopathy [20] or atypical scrapie [33] may be easier to faithfully replicate via *in-vitro* amplification methods. TSE strains are known to exist as numerous distinct disease entities, more than one of which can co-exist in a single host [9], [10], [24], [34]. The presence of more than one TSE strain in individual sheep or mice has previously been demonstrated suggesting that strain competition, where one strain prevails over another, is not absolute [9], [10], [24]. Strain competition or interaction can also occur during PMCA and may have as yet unknown effects on the PMCA product. Indeed Shikiya et al. [16] demonstrated that the ‘drowsy’ strain of hamster adapted transmissible mink encephalopathy could interfere with the alternative ‘hyper’ strain during *in-vitro* amplification (PMCA) as well as *in-vivo* transmissions, possibly due to competition or sequestration of PrP^C^ or other cellular co-factors. However, since that study determined the presence of resultant strains by the migration properties of PrP^Sc^ on Western blot, the biological phenotype of such mixtures is unknown. In an *in-vivo* system, as yet unknown accessory molecules, processes or genetic factors that govern replication may be involved that are not represented within an *in-vitro* system. Whilst this may not preclude infectivity of the *in-vitro* generated product it may influence its biochemical properties and ultimately affect the phenotype of the resultant product upon bioassay. To this effect a previous study demonstrated that infectious hamster prions could be generated by PMCA using a substrate containing only PrP^C^, co-purified lipid molecules and accessory polyanion molecules, possibly acting as catalysts or scaffolds complexed to PrP^Sc^ [30]. However, the resultant phenotype following inoculation into hamsters differed to that of the original seed.

In conclusion, of the two field scrapie sources investigated, the phenotypes of one source on transmission to mice appear to have been faithfully replicated by PMCA whilst the other has produced a phenotype which does not directly compare with those that arose through traditional bioassay. These data suggest that differences in the efficiency of PMCA as a strain-typing tool to replace primary passage in mice may vary between classical scrapie isolates and therefore the technique cannot be reliably used in the strain-typing of classical scrapie field sources. It is possible that certain scrapie strains may be more permissible to faithful replication by PMCA than others, perhaps determined by their speed or propensity to convert at a conformational level or both. PMCA may prove more reliable with TSEs attributed to single strains such as classical BSE or atypical scrapie. Since there are significant potential benefits of using PMCA as a strain typing tool, particularly in terms of reducing animal numbers and time required for strain typing, further research should be carried out to determine the capacity in which PMCA can be reliably used for high fidelity amplification of strains arising from different TSE sources.
